# Bright emission from a random Raman laser

**DOI:** 10.1038/ncomms5356

**Published:** 2014-07-11

**Authors:** Brett H. Hokr, Joel N. Bixler, Michael T. Cone, John D. Mason, Hope T. Beier, Gary D. Noojin, Georgi I. Petrov, Leonid A. Golovan, Robert J. Thomas, Benjamin A. Rockwell, Vladislav V. Yakovlev

**Affiliations:** 1 Department of Physics & Astronomy, Texas A&M University, College Station, Texas 77843, USA; 2 Department of Biomedical Engineering, Texas A&M University, Texas 77843, USA; 3 711th Human Performance Wing, Human Effectiveness Directorate, Bioeffects Division, Optical Radiation Bioeffects Branch, JBSA Fort Sam Houston, Texas 78234, USA; 4 TASC Inc., San Antonio, Texas 78228, USA; 5 Faculty of Physics, M. V. Lomonosov Moscow State University, Moscow 119991, Russia

## Abstract

Random lasers are a developing class of light sources that utilize a highly disordered gain medium as opposed to a conventional optical cavity. Although traditional random lasers often have a relatively broad emission spectrum, a random laser that utilizes vibration transitions via Raman scattering allows for an extremely narrow bandwidth, on the order of 10 cm^−1^. Here we demonstrate the first experimental evidence of lasing via a Raman interaction in a bulk three-dimensional random medium, with conversion efficiencies on the order of a few percent. Furthermore, Monte Carlo simulations are used to study the complex spatial and temporal dynamics of nonlinear processes in turbid media. In addition to providing a large signal, characteristic of the Raman medium, the random Raman laser offers us an entirely new tool for studying the dynamics of gain in a turbid medium.

## Abstract (web summary)

Unlike conventional lasers that require a uniform resonant cavity to operate, random lasers use a highly disordered gain medium in which scattering is dominant. Hokr *et al*. report Raman lasing from a bulk three-dimensional disordered medium whose intensity exceeds that of other random lasers by many orders of magnitude.

## Graphical abstract

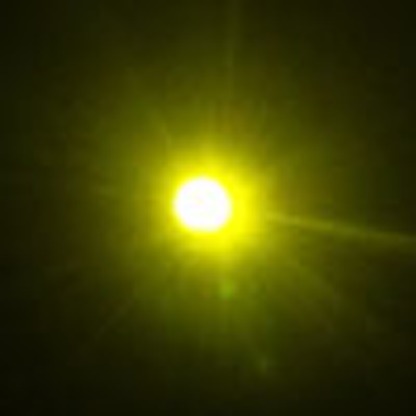

The propagation of light in a turbid medium is something that everyone experiences on a daily basis, yet there are many related fundamental problems that are left to be understood^1^. Everything from climate change^2^, to biological imaging^3^, to defending against terrorism attacks^4^ benefit from a deeper understanding of how light propagates through a random medium. Throughout the last three decades numerous new phenomena, facilitated by elastic scattering, have been discovered and explored, such as coherent backscattering^5^,^6^, focusing via wavefront optimization^7^,^8^, and random lasing^9^,^10^. Conventional wisdom suggests that nonlinear effects, such as stimulated Raman scattering (SRS), should not have a significant role in the propagation of light through random media. The diffusive nature of elastic scattering restricts the interaction distance by limiting the depth at which high intensities can be delivered, thus reducing the efficiency of nonlinear optical effects. However, light scattering can dramatically increase the interaction length by multiply scattering the photons in a random walk type motion, making the overall outcome somewhat hard to predict. An understanding of these dynamics are especially important for deep-tissue optical imaging utilizing multiphoton fluorescence^11^,^12^ and SRS^13^,^14^.

Raman scattering is the inelastic scattering of a photon from a molecule. The frequency of the scattered photon is determined by the frequency of a vibrational level of the molecule. The frequency shift of the Raman light with respect to the pump light is unique to the molecule, making Raman spectroscopy a valuable tool for molecular and structural identification^15^. In the context of Raman lasing, spontaneous Raman scattering and SRS are analogous to fluorescence and stimulated emission, respectively. However, the nonlinearity associated with SRS distinguishes it from stimulated emission^16^. The Raman gain is proportional to the intensity of the pump. Unlike traditional lasing, energy cannot be stored by the system after the pump pulse is gone^17^,^18^,^19^,^20^. Thus, to observe Raman lasing, the medium must be pumped hard and fast. Furthermore, Raman lasers and traditional lasers differ in that the wavelength of light required to pump the laser does not depend on the electronic structure of the medium, and thus can be chosen to minimize absorption.

Random lasing was first predicted by V. S. Letokhov in 1968 (ref. 10), but was not experimentally observed until 1994 (refs 21, 22). Random lasers operate on many of the same principles as traditional lasers, except that multiple elastic scattering provides feedback in place of a Fabry–Perot cavity. Analogous to traditional lasers, random lasers have a threshold where gain exceeds losses and exponential amplification occurs at the lasing frequency^23^. Random lasers are traditionally divided into two categories: coherent and incoherent, based on the feedback that drives them^24^. Coherent feedback occurs with the generation of unstable periodic trajectories by multiple elastic scattering events^25^. These trajectories exhibit modes which are analogous to many randomly oriented ring cavities existing in the gain medium. When multiple scattering acts only to return energy back into the gain medium, but phase information is lost in the process, the system is driven by incoherent feedback^9^. Compared with the media typically used in random lasing experiments, SRS has a narrow gain bandwidth (typically less than 10 cm^−1^). Thus, it is unlikely that a random lasing mode will lie in the gain bandwidth, making incoherent feedback the more likely dominant mechanism in random Raman lasing.

A random Raman laser uses SRS as the primary gain mechanism and relies on elastic scattering to provide feedback into the gain medium. To date, random Raman lasing has only been observed in a one-dimensional fibre system^26^. From a random walk viewpoint, one-dimensional systems are fundamentally different from three-dimensional systems. In a random walk of two or lower dimensions in an infinite space, with infinite time, the walker will eventually return to the starting location. However, in three dimensions there is no guarantee that the walker will ever return. This leads to a fundamental difference in the dynamics of photon diffusion in three dimensions.

Here we demonstrate the first experimental evidence of lasing via a Raman interaction in a bulk three-dimensional random medium. The complicated dynamics of nonlinear pulse propagation in a turbid medium make an analytical approach to describing this problem as very challenging. To better understand these processes, an earlier introduced^27^,^28^ Monte Carlo model was employed. These simulations provided a guide to the experiments and illuminated aspects of the dynamics that cannot be easily observed experimentally.

## Results

### Experimental setup

**Figure 1:**
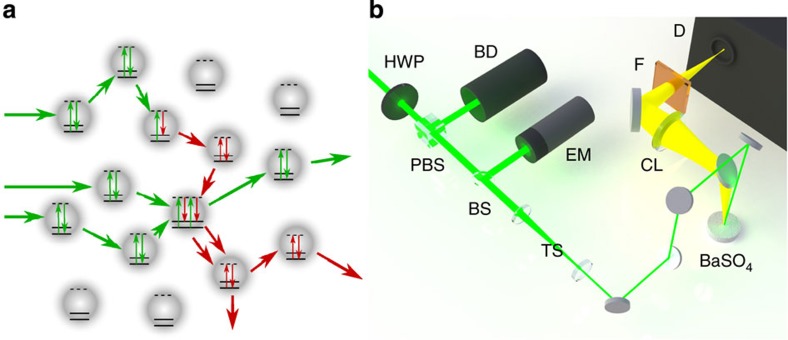
Conceptual figure of random Raman lasing and experimental layout. (**a**) Conceptual drawing illustrating random Raman lasing that is built up from spontaneous Raman scattering. Several pump photons are shown entering the medium. One undergoes spontaneous Raman scattering whereas the other two are simply elastically scattered. An additional Raman photon is created via stimulated Raman scattering between the two elastically scattered pump photons and the Raman photon. This is the mechanism that drives random Raman lasing. (**b**) Simplified diagram of the experimental setup for the random Raman laser. BaSO_4_, barium sulphate powder with micron-sized particles; BD, beam dump; BS, beam splitter; CL, collection lens set up to image the sample onto the detector; D, either an energy metre, spectrometer or streak camera depending on the desired measurement; EM, energy metre; F, filter; HWP, half-wave plate; PBS, polarized beam splitter, TS, slightly offset × 3 telescope to gently focus the beam onto the sample.

The random Raman laser, illustrated in Fig. 1a, was made of barium sulphate (BaSO_4_) powder with particle diameters of 1–5 μm. BaSO_4_ has the role of both the Raman gain medium and the scattering centres. BaSO_4_ was chosen due to its low absorption, both linear and nonlinear, and high scattering cross section throughout the visible spectrum. To pump the random Raman laser, short laser pulses with a centre wavelength of 532 nm and a pulse duration of 50 ps were used. The incident radiation was gently focused onto the sample using a slightly offset beam expander as a compound lens, allowing for adjustments in the beam diameter incident on the sample (see Fig. 1b). The best results were achieved with a beam diameter of about 1 mm. Smaller beam diameters limited the maximum amount of energy which could be deposited without observing damage to the sample, whereas the efficiency declined due to lower intensities when larger beam diameters are used. The emitted light was collected at near normal incidence and passed into an energy metre, a CCD camera or a spectrometer via a set of mirrors and lenses for the appropriate measurements.

### Threshold dynamics and efficiency

**Figure 2:**
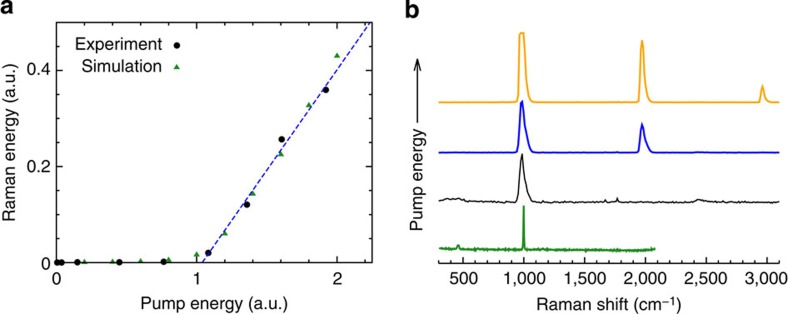
Threshold behaviour of random Raman lasing. (**a**) Output Raman pulse energy versus incident pump pulse energy indicating a clear threshold where random Raman lasing begins. Black circles are the experimental data points, dashed blue line is a linear fit of the last four experimental data points and green triangles are the results of Monte Carlo simulations. (**b**) SRS spectra of BaSO_4_ powder at various pump powers illustrating the presence of higher-order Raman transitions. The spontaneous Raman spectrum of BaSO_4_ is shown for comparison, but was taken with a much higher resolution spectrometer. All spectra have been normalized to fit on the same scale, and the widths of the SRS spectral peaks were limited by the resolution of the spectrometer used. a.u., arbitrary unit.

At low pump pulse energies, spontaneous Raman scattering, which manifests itself as a spectrum consisting of several lines whose relative intensities are not affected by the pump intensity, dominates the detected signal. Above the lasing threshold, the Raman spectrum collapses to a single Raman peak with a frequency shift of 985 cm^−1^ as shown in Fig. 2b. At higher pump energies, a second and third peak were observed corresponding to the second- and third-order Stokes signal. These higher-order signals are the result of light undergoing multiple shifts of 985 cm^−1^. For example, the second-order Stokes signal is generated by the first Stokes light undergoing a second SRS process in which it picks up an additional 985-cm^−1^ frequency shift, resulting in a signal with a total frequency shift from the fundamental pump wavelength of 1,970 cm^−1^, and so on for the higher-order processes. The presence of these higher-order processes further illustrate the substantial efficiency of the random Raman lasing process.

**Figure 3:**
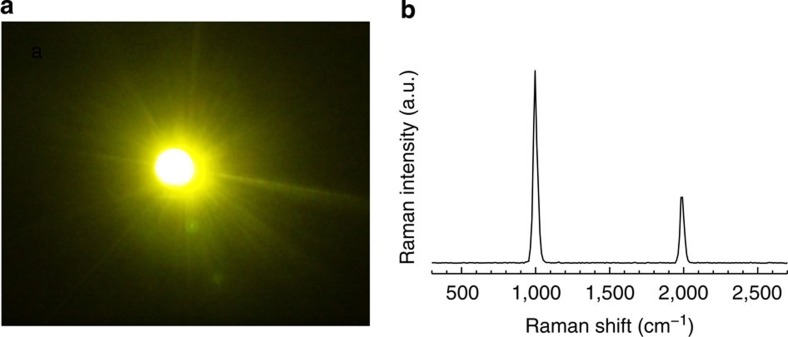
Bright emission from random Raman lasing. (**a**) Digital camera photo of random Raman lasing in BaSO_4_ powder. (**b**) Spectrum of BaSO_4_ taken through a 20.3 cm collection optic, 21 m away from the sample. a.u., arbitrary unit.

Once the pump energy increases beyond the threshold, (1.05 mJ in the experiment) gain exceeds losses and SRS dominates the conversion process and random Raman lasing ensues (see Fig. 2a). At a maximum of 11.5 mJ of pump energy, 2.0 μJ of Raman signal was measured by the energy metre. Assuming a homogeneous angular distribution of the emitted Raman photons, the conversion efficiency of pump photons into Raman photons was approximately 1%. The astounding brightness of the random Raman laser is illustrated in Fig. 3 with a digital photograph and with a spectrum taken from a distance 21 m from the sample using a 20.3 cm off-axis parabolic mirror as a collection optic. This demonstrates that the random Raman laser is remarkably efficient considering typical conversion efficiencies for spontaneous Raman scattering are on the order of 10^−8^ (ref. 29).

**Figure 4:**
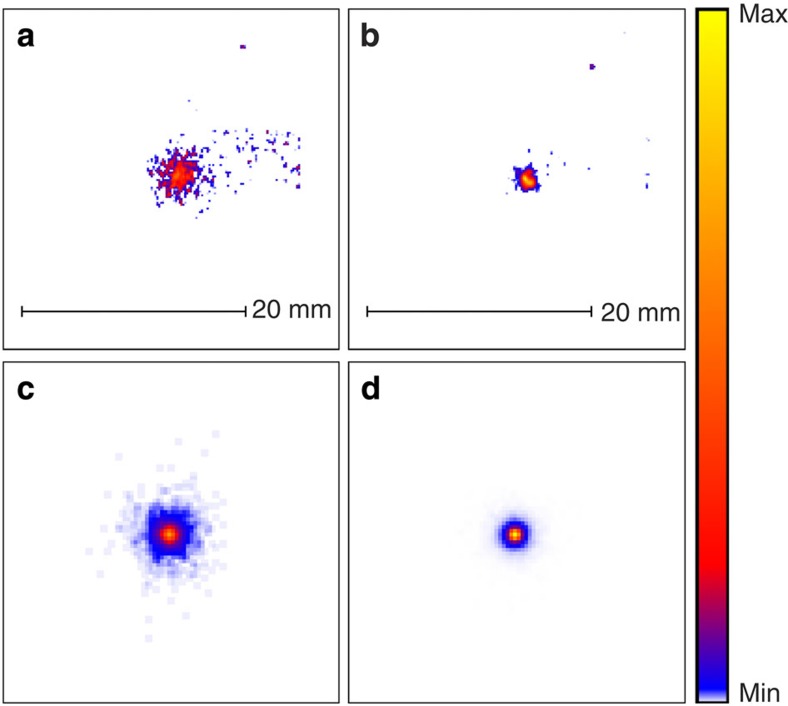
Threshold behaviour of the spatial distribution. Spatial beam profiles, (**a**) below threshold experiment, (**b**) above threshold experiment, (**c**) below threshold simulation and (**d**) above threshold simulation illustrating the significant change in the spatial profile of the Raman signal above and below the threshold. Background subtraction was preformed for the experimental images.

Furthermore, a distinct feature of random Raman lasing is found in the dramatic variation of the beam profile of the emitted radiation on the surface of the sample. Below the lasing threshold, the spatial distribution of the emitted light of the random Raman laser is very broad and is due to spontaneous Raman scattering (see Fig. 4). This is the result of deep-penetrating photons which spend a longer time in the medium, leading to a greater chance of inelastic scattering through Raman processes^27^. These deep-penetrating photons have a larger probability of exiting the sample with large radial offsets. However, above the threshold, the majority of the energy is emitted from a highly localized area near the surface (see Fig. 4). This is primarily due to the fact that a large amount of the SRS light is generated close to the surface, resulting in much smaller radial offsets. Strong SRS generation near the surface can be attributed to the slower speed of light intensity in turbid media compared with vacuum^30^,^31^. Thus, photons initially arriving in the medium do not have time to leave the front surface of the sample before photons from the back of the pulse enter, causing the pulse to effectively be compressed. This light compression raises the intensity, generating a large SRS signal close to the surface (See Supplementary Movie 1).

## Discussion

Many properties of the random Raman laser closely mimic those of a traditional random laser, however, there are some notable differences. First, the gain bandwidth of a Raman transition is quite narrow (on the order of several cm^−1^) and as a result, Raman lasing is monitored by measuring the relative increase of intensity of the strongest Raman line. Second, non-resonant Raman transitions involve virtual states, and are much faster than electronic transitions involving real atomic or molecular levels. Thus, transient (picosecond) dynamics of the pump pulse propagation through the medium have a pivotal role.

In our experiment, micron-sized particles were chosen to ensure that no single particle promoted significant Raman gain^32^,^33^, thus requiring the feedback from elastic scattering to support random Raman lasing. When larger particles (hundreds of microns or larger) are used, it is possible that a single particle can support substantial Raman gain. This Raman signal would then be scattered around and would mimic random Raman lasing, however, it should be stressed that this would not be random Raman lasing. Such a situation would result in ordinary Raman lasing in a single particle which is then diffused throughout the medium by elastic scattering without subsequent amplification occurring.

We have demonstrated the first random Raman laser in a bulk three-dimensional random medium. The lasing process is characterized by a distinct threshold, which is well below the damage threshold of the materials used for these studies. Although many of the features are similar to traditional random lasers, the dynamics of random Raman lasing have a few notable differences, which we have elucidated with the help of Monte Carlo simulations. Although this report was focused on barium sulphate, the same effect has been observed in a number of organic and inorganic powders, using both picosecond and nanosecond laser pulses.

## Methods

### Experimental setup

For the pump source, the second harmonic pulse out of a Spectra Physics Quanta-Ray GCR-3RA that was injection-seeded with a 10-ps pulse out of a Spectra Physics Vanguard HM532 was used. This produces a 50-ps pulse at 532 nm. To control the pump power a half-wave plate was followed by a polarizing beam splitter. This allows us to adjust the intensity of the pumping laser without effecting the beam quality. The pump pulse is then gently focused onto the 1–5 μm BaSO_4_ powder that was loosely packed into a small Petri dish by using a slightly offset × 3 telescope consisting of a 150-mm focal length plano-convex lens, followed by a 50-mm focal length plano-concave lens. This setup allows us to slightly tune the spot size on the sample while maintaining a nearly collimated beam. The Fresnel reflections off of a 1.6-mm thick BK7 window, tilted at 45° from the beam axis, was used as a reference signal to measure the pump power. This signal was detected via a Coherent J4-09 energy metre. The reference signal was calibrated by measuring the ratio between the energy of the reference pulse and the energy of the pump pulse at the sample, using an additional Coherent J4-09 energy metre.

The output Raman signal was picked off using a nearly parallel elliptical mirror, and imaged onto the detecting apparatus using a 5.08-cm diameter, 125-mm focal length lens. The excess 532-nm light was removed by using both lenses of a pair UVEX L99-LS6 YAG/KTP laser safety glasses as filters. The two lenses combined to give 9.00 OD of attenuation at 532 nm, 0.51 OD at 563 nm, and 0.47 OD at 594 nm. These absorption measurements were made using a Cary 5 G spectrophotometer. These wavelengths correspond to the pump, the first-order SRS peak at 985 cm^−1^, and the second-order SRS peak at 1,970 cm^−1^, respectively. Using an Ocean Optics USB 2000 spectrometer, it was confirmed that these filters reduce the 532-nm light to a level below that which can be measured by that spectrometer. For measuring the Raman energy in Fig. 2a a Coherent J3S-05 energy metre was used for the detecting apparatus. To measure the SRS spectra shown in Fig. 2b, an Ocean Optics USB 2000 spectrometer was used. For the spontaneous Raman spectrum, an InSpectrum 300-mm spectrometer (Acton Research, Inc.) was used.

The spatial profiles shown in Fig. 3a,b were obtained using the same laser system, but the setup differed slightly. Instead of having the powder in a Petri dish and shining the laser down from the top, the BaSO_4_ powder was placed in a 2 cm by 1 cm cuvette and imaged from the front using a Pulnix TM-6701AN monochrome CCD camera.

For the 21-m spectra, a 20.3-cm off-axis parabolic mirror with a focal length of 2.54 m, was set up across the lab at a distance of 21 m from the sample. A 5.08-cm diameter 193-mm focal length achromatic lens was placed near the focal spot of the mirror, 145 mm in front of the fibre mount of an Ocean Optics USB 2000 spectrometer, to further focus the signal onto the slit, but without collecting any additional signal.

### Monte carlo simulations

For spontaneous Raman scattering, a Monte Carlo model developed previously was used^27^,^28^. We will quickly review the model here for clarity. A Gaussian pulse, consisting of 10^5^ photons is sent into a scattering medium characterized by scattering (*l*_*s*_), absorption (*l*_*a*_) and Raman (*l*_*R*_) mean free paths. Photons are propagated for a fixed amount of time during each step of the simulation. This is in contrast to many Monte Carlo schemes that propagate each photon by the distance to its next scattering event, thus causing all photons in the simulation to effectively have different clocks. During each step there is a probability of an event given by


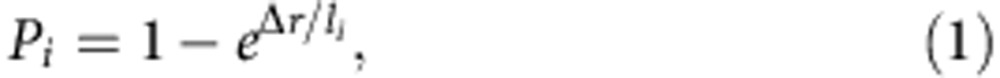


where i represents elastic scattering, absorption or Raman scattering, and Δ*r*=(*c*/*n*)Δ*t*. Elastic scattering is assumed to be anisotropic and is described by the Henyey–Greenstein probability distribution, characterized by the anisotropy factor *g*=‹cosθ›. Spontaneous Raman scattering is assumed to be anisotropic.

To include SRS effects, an interaction between a pump and Raman photons is defined. For each Raman photon inside a sphere of radius *r*_SRS_, centred around the pump photon, there is a binomial probability of converting that pump photon into a Raman photon given by


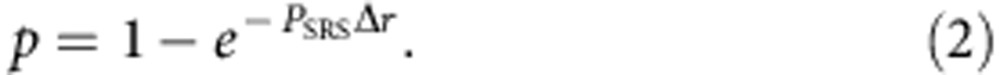


Here, *P*_SRS_ is a probability per length used to describe SRS and is related to the Raman gain coefficient, *G*. When a pump photon is converted, the new Raman photon takes the direction of the Raman photon whose coin flip generated the conversion.

For all the simulations presented in this paper the following values for the parameters were used: *l*_*s*_=0.005 mm, *l*_*a*_=10.0 mm, *l*_*R*_=200 mm, *r*_SRS_=0.025 mm, *P*_SRS_=0.1 mm^−1^, *n*=1.6, *g*=0.6, Δ*r*=0.002 mm. The sample was assumed to be 5 mm thick and infinite in the transverse directions to simulate an infinitely thick medium (no photons were transmitted through the sample in any run). The incident pulse was assumed to be a 40-ps full-width at half-maximum pulse with a full-width at half-maximum beam diameter of 0.1 mm To generate the data in Fig. 2a, 25 independent runs were averaged, whereas Fig. 3c,d were generated using only a single run. To ensure that SRS did not contribute to the below threshold data presented in Fig. 3c, stimulated effects were disabled in this simulation.

Supplementary Movie 1 was run with the same parameters as above. The black box is drawn for scale and perspective, and is not a physical boundary. It measures 1 cm by 1 cm in the transverse directions and is 0.5 cm deep (corresponding to the depth of the simulation). Green spheres of radius *r*_SRS_ are drawn to indicate photons at the pump wavelength. Likewise, red spheres are drawn to indicate the Raman scattered photons. The videos on the left and right are of the same simulation, but the video on the left draws both the pump and Raman photons, whereas the video on the right draws only the Raman photons.

## Author contributions

B.H.H., J.N.B., M.T.C., J.D.M., H.T.B., G.D.N., G.I.P., B.A.R. and V.V.Y. conducted experiments. B.H.H. and R.J.T. conducted simulations. B.H.H., B.A.R., G.I.P., L.A.G. and V.V.Y. designed research. All authors contributed to the preparation of this manuscript.

## Additional information

**How to cite this article:** Hokr, B. H. *et al*. Bright emission from a random Raman laser. *Nat. Commun.* 5:4356 doi: 10.1038/ncomms5356 (2014).

## Supplementary Material

Supplementary Movie 1 Monte Carlo simulation depicting the formation and dynamics of random Raman lasing. Both the left and the right side of the video are from the same simulation, but the left side shows both the pump and the Raman photons, while the right side shows only the Raman photons. The simulated time is shown in the lower left hand side of the video.
